# Revaccination

**Published:** 1858-01

**Authors:** 


*Revaccination.*—We see from the French Journals that this subject is being agitated in France. The *Académie de Médecine* has re-commenced a system of Revaccination to be enforced by law. One of the great causes of failure in vaccination, is the difficulty of preserving the vaccine matter. All agree in the impossibility of preserving it between tied pieces of glass.

